# Supplementary material for: Deciphering SCN2A: A comprehensive review of rodent models of Scn2a dysfunction
Source: ArXiv. 2024 Nov 15:arXiv:2411.10421v1. Preprint. [Version 1] (PMC11601800)
Supplement: Supplement 1 [file NIHPP2411.10421v1-supplement-1.pdf]

Model	Principle Paper	Related Papers	Iteration of Conditional Model
**Constitutive Haploinsufficient Mouse Models of SCN2A**			
Scn2a +/-	Planells-Cases et al., 2000	Planells-Cases et al., 2000	
		Mishra et al., 2017	
		Middleton et al., 2018	
		Ogiwara et al., 2018	
		Lena and Mantegazza 2019	
		Spratt et al., 2019	
		Tatsukawa et al., 2019	
		Miyamoto et al., 2019	
		Spratt et al., 2021	
		Indumathy et al., 2021	
		Tamura et al., 2022	
		Schamiloglu et al., 2023	
		Wang et al., 2024	
		Marcantonio et al.,2023	
Scn2a+/–; Kcna1-/-	Mishra et al., 2017	Mishra et al., 2008	
		Indumathy et al., 2021	
Scn2a+/–; Kcna1+/–	Indumathy et al., 2021		
NaV1.2 adult	Gazina et al., 2015		
Scn2a fl/+	Shin et al., 2019		
Scn2a +/KI	Tamura et al., 2022	Tamura et al., 2022	
**Premature Stop Codon Models**			
Scn2a R102X/+	Ogiwara et al., 2018		
**Conditional Mouse Models of Scn2a Haploinsufficiency**			
Ei14::Scn2a+/-	Spratt et al., 2019	Spratt et al., 2019	PV-Cre
			SOM-Cre
Scn2a +/fl			CaMKIIα-Cre
		Spratt et al., 2021	EF1α-Cre (Scn2a +/fl and Scn2a fl/fl)
		Wang et al., 2024	Alpha6-Cre
SCN2A f/f	Gould and Kim 2021		PDGFRA - Cre
			Actin- Cre
Scn2a fl/+	Yamagata et al., 2017		Vgat-Venus
	Ogiwara et al., 2018	Ogiwara et al., 2018	Emx1-Cre
			Vgat-Cre
			Vgat-Venus
		Tatsukawa et al., 2019	Emx1-Cre
			Vgat-Cre
		Miyamoto et al., 2019	Emx1-Cre
			Vgat-Cre
Scn2a fl/fl		Miyamoto et al., 2019	Trpc-Cre
			Ntsr-Cre
		Suzuki et al., 2023	mPFC-Cre
			VTA-Cre
Scn2a +/KI	Tamura et al., 2022		EF1α-Cre
Scn2a fl/fl	Ma et al., 2022		SCN-Cre
**Additional Mouse Models of Scn2a Reduction**			
Scn2a :OB GC - shRNA	Nunes and Kuner, 2018		
Scn2aΔ1898/+	Wang et al., 2021		
Scn2a gtKO/gtKO	Eaton et al., 2021	Eaton et al., 2021	
		Ma et al., 2022	
		Wu et al., 2024	
Scn2a ASO	Li et al., 2023		
**SCN2A Gain of Function Mouse Models**			
Scn2a Q54	Kearney et al., 2001	Kearney et al., 2001	
		MacDonald et al., 2004	
		Bergren et al., 2005	
		Bergren et al., 2009	
		Kile et al., 2008	
		Anderson et al., 2014	
		Kim et al., 2016	
		Calhoun et al., 2016	
		Hawkins & Kearney, 2016	
		Thompson et al., 2017	
Scn2a A263v /+	Schattling et al., 2019		
Scn2a Q/+	Li et al, 2021		
**Other SCN2A Mouse Models**			
Scn2a E/+	Echeveria-Cooper et al., 2022	Echeveria- Cooper et al., 2022	
		Echeveria- Cooper et al., 2023	
		Echeveria- Cooper and Keanrey, 2023	

Paper	*Model*	Iteration of Conditional Model	Mice Age	Social	Learning/Memory	Motor	Repetitive Behaviors	Anxiety/Hyperactivity	Olfaction	Seizures	Other
**Planells-Cases *Scn2a +/-* Mouse Model**											
Mishra et al., 2017			2 - 3 months							-ND in seizure threshold or epiliptiform activity - tency to myoclonic jerk -ND in latency to GTC seizures	-ND in cardiac activity with EEG-ECG ecordings -ND in brain-heart association
Middleton et al., 2018 **(males only)**			4 - 6 months		**-Delayed learning in spatial working memory task - Slower learning in Barnes maze**						**-Decrease in the peak firing rate of yramidal cells during SPW-R events -Lower strength of assembly luring SPW-Rs -Truncated hippocampal replay sequences**
Ogiwara et al., 2018			6 - 11 weeks							-ND in susceptibility to ia induced seizures **-Frequently abnormal ECoG recordings with absence- with SWDs -Higher maximum amplitude and spike numbers during a VD episode -Shorter latency to have absence seizure-like immobility when injected with PTZ**	**-Higher prevalence and incidence of epileptiform discharges in mPFC -Impaired excitatory neural activity**
Lena and Mantegazza, 2019 **(males only)**			Juvenile: P22 - P44	**-Decreased amount and length of USVs produced -Decreased social interaction in the first two minutes of reciprocal social interaction test**	**-Slight decrease of spontaneous alternations in Y maze - Decreased preference for novel object in NOR task**	**-Increased acquired motor learning on rotarod**	**ncreased grooming -Increased marble burying**	**-Reduced anxiety in EPM - Reduced anxiety in OFT** - ND in locomotion in OFT	-ND in odor discrimination task		**-Decreased immobility in TST**
			Adult: P60 - P95	**-Decreased amount and length of USVs produced** -ND in reciprocal social interaction test	-ND in number of visits or spontaneous alternations in Y maze - ND in preference for novel object in NOR task - **Slightly slower acquisition latency in Barnes maze test**	-ND in motor learning in rotarod	-ND in grooming - ND in marble burying	-ND in anxiety in EPM - ND in anxiety in OFT - ND in locomotion in OFT	-ND in odor discrimination task		**-Slight decrease in immobility in minutes four to six of TST**
Spratt et al., 2019			Age not reported	**-Females showed no preference for novel stimuli in two-chamber social task**	**-Males had slightly impaired reversal learning water T-maze** -ND in spontaneous alternation assay	-ND in rotarod - ND in balance beam	-ND in grooming - ND in nesting assay	**-Females had reduced xiety in EPM** -ND in OFT			
Tatsuwaka et al., 2019 **(males only)**	*Scn2a +/- (Planells-Cases model)*		*P6 for USV *8 - 31 weeks for other assays	**-Increased sociability in e-chamber assay -Slight dominant behavior n tube test -Deficit in social approach toward stranger mice in resident-intruder test** - ND in social communication in USV	**-Enhanced fear nditioning retention -Impaired exctinction of fear-related memory**	**-Deficient motor coordination in rotarod**	**-Increased rearing in OFT**	**duced anxiety in EPM tivity in EPM duced anxiety in OFT activity in OFT -Increased anxiety in light/dark assay**			**-Higher immobility in FST immobility in TST** -ND in PPI **-Increased activity in the gamma band in the mPFC**
Miyamoto et al., 2019			> 8 weeks							**-Have SWDs during behavioral quiescence**	**-Slight decrease in EPSCs in FSIs**
Indumathy et al., 2021			28 - 34 days	-ND in sociability in three-chamber assay - **Decreased preference for novel stranger mice in social novelty assay**			**-Reduced marble burying - Increased grooming**				-ND in nestlet shredding
Schamiloglu et al., 2023			P54 - P119		-ND in learning the standard dynamic foraging task or variations of the assay where mice are head-fixed, the block length varies, there is reward optimization, or the ies vary -Slight increase in frequency to pick higher reward side in ng assay **-Males had inflexible choice behavior during mid-block performance in the unpredictable paradigm of the dynamic foraging assay** - ND in intertrial interval in dynamic foraging assay					**-Spontaneous behavioral seizures in four of twenty five mice at the end of a day's training and one of the four mice died**	
Wang et al., 2024			P60 - P90								**-Consistently elevated VOR gain in different rotation frequencies -Decreased Purkinje cell firing rate**
			Juvenile: P21 - P44	**-Higher sociability in ree-chamber assay** -ND in social novelty in three-chamber assay	-ND in preference for displaced object in novel gnition task **-Lower entries into the of the Y maze -Increased decision-making latency in Y maze**	**-Higher latency to fall on day one of rotarod**	-ND in grooming - ND in marble burying	-ND in EPM locomotion - **Reduced anxiety in EPM** - ND in anxiety in OFT - ND in OFT locomotion	-ND in odor discrimination task		-ND in latency of reaction to stimuli in hotplate assay - ND in latency before withdrawal in coldplate asaay

RED = Decrese
BLUE= Increase/altered
ND= No Difference

OFT = Open Field Test
EPM = Elevated Plus Maze
FST = Forced Swim Test
TST = Tail Suspension Test
USV = Ultrasonic Vocalization
PPI = Prepulse Inhibition
mPFC = Medial Prefrontal Cortex
ECoG = Electrocorticogram
PTZ = Pentylenetetrazol
FSI = fast-spiking interneurons
EPSCs = excitatory postsynaptic currents
VOR = Vestibular Ocular Reflex

Paper	*Model*	Iteration of Conditional Model	Mice Age	Social	Learning/Memory	Motor	Repetitive Behaviors	Anxiety/Hyperactivity	Olfaction	Seizures	Other
Marcantonio et al., 2023 **(females only)**			Adult: >P60	**-Higher sociability in ree-chamber assay -Altered interaction with novel mouse in three-chamber assay**	-ND in preference for ct in NOR task **-Lower entries into the three arms of Y maze - Increased decision-making atency in Y maze -Reduced percent of spontaneous alternations in Y maze**	-ND in latency to fall in rotarod	-ND in grooming - ND in marble burying	-ND in EPM locomotion - ıced anxiety in EPM -ND in anxiety in OFT - ND in OFT locomotion	-ND in odor discrimination task		-ND in latency of reaction to stimuli in otplate assay **-Decreased latency before withdrawal in coldplate**
**Other Constitutive Haploinsufficient Mouse Models Behavior**											
Mishra et al., 2017	*Scn2a+/-; Kcna1-/-*		2-3 months							**Scn2a+/– decreases spontaneous seizure duration in Kcna1–/– mice**	Nav1.2 reduction does not ameliorate cardiac phentoypes in Kcna1–/– mice.
Indumathy et al., 2021			28-34 days	-ND in sociaialbility **- Reduced social novelty sniffing**			rooming **-Reduced nestlet shredding and marble burying**				
	Scn2a+/–; Kcna1+/–			ND in socialbility or social novelty			-ND in grooming and nestlest hredding **-Reduced marble burying**				
Gazina et al., 2015 **(males only)**	*NaV1.2 adult*		53 days >=	-ND in social interaction **-16x more likely to jump out of social interaction box**	ND in memory and spatial learning on Y-maze and Morris water maze	ND on rotarod, locomotion or in grip strength		-ND in OFT or Light-Dark box **Reduced anxiety in EPM**		ontaneous seizures **- More susceptible to PTZ-induced seizures**	ND in thermal pain response, acoustic startle, PPI, or weight
Shin et al., 2019 **(males only)**	*Scn2a f/+*		2-4 months	-ND social approach and communication - **Increased direct social interation**	**-Reduced Spatial learning and memory on Morris ater maze -Increased Fear memory**	D on Rotarod -ND in locatmotion in novel enviroments **-Decreased locomotion in familar enviroments**	ND in grooming and rearing behaviors	-ND in anxiety for OFT, EPM and Light-Dark box - **Mild light induced hypoactivity**		ontaneous seizures -ND in susctpibility to PTZ-induced seziures	ND for novel object recognition
			4 days - 3 weeks	-ND in number and duration of USVs **-Slight increase direct social interaction**		**Decreased locomotion OFT**	ND in grooming	ND OFT			ND in mother attachment behaviors
**Hypomorphic *Scn2a* Mouse Model Behavior**											
Wang et al., 2021	*Scn2a Δ1898/+*		>= 30 to 44 days	**-Males displayed increased social interacton with novel mouse in 3-chamber - After repeat exposure to the same mouse, males , still displayed increased social interaction**		ND in general locomotion	ND in time spent grooming	**ty in OFT - Reduced anxiety-like behaviors on EPM**			
Eaton et al., 2021	*Scn2a gtKO/gtKO (AKA Scn2a Trap)*		3 to 5 months		**-Decreased novel object -Increased fixed-pattern of exploration in novel object -Decreased number of entries to, duration in, and prefrence for the novel arm of Y-maze**	-ND activity, tail posture, limb grasping, extension, ghting reflex **-Decreased grip ability n SHIPRA -Females traveled longer -Decrease in distance traveld in OFT perfromed with increased birightness**	**-Decreased overall nest building - Decreased quaility of nests built - Increased grooming - d marble burying -Slightly increased induced ng**	**-Increased anxiety-like behavior in low and bright ght OFT** -ND freezing behavior in low FT **- Increased freezing in OFT perfromed with high intensity lumnesience**	-ND overall odor discrimination - **Increased in time for discrimination**		**eased water and food intake r of fecal boli in males -Lower body position rating, less trunk curl and postural reflex when held by SHIPRA -Decresed tolerance for heat and cold temperatures on thermal/cold ssay** -ND for dynamic hot plate Increased e ce **-Decreased startle response in PPI**
Ma et al., 2022			> 6 weeks			**Impairment of wheel-running activity in light-dark conditions**					**Abnormal sleep patterns**
Wu et al., 2024			3 to 4 months		**Impaired learning and memory on Borris water maze and Y-maze**						
**Conditional and other Mouse Models of *Scn2a* Insufficiency**											
Wang et al., 2024	*Scn2a +/fl*	Alpha6-Cre	60 to 90 days								**Altered VOR**
Tatsukawa et al., 2019	*Scn2a fl/+*	Emx1-Cre	8 to 9 weeks **(Male Only)**			**Increased rearing in OFT**		**-Increased time in center of FT -Trend of entering open arms of EPM more**			
		Vgat-Cre				ND in locomotion or rearing		-ND OFT - **Reduced anxiety-like behaviors on EPM**			
Miyamoto et al., 2019		Emx1-Cre	>= 8 weeks							**-SWDs present during ehavioral quiescence -Inactivation of the CPu or thalamus by muscimol injection suppresses SWDs**	
		Vgat-Cre								No spontaneous SWDs	
	Scn2a fl/fl	Trpc-Cre								**Spontaneous SWDs**	
		Ntsr-Cre								No spontaneous SWDs	
Ma et al., 2022	*Scn2afl/fl*	SCN-Cre	3-5 months								**Sleep Disturbances**
Suzuki et al., 2023	*Scn2a fl/fl*	mPFC-Cre	18 to 19 weeks	**Increased sociablity in three chamber social approach**		**-Less distance traveld in hamber social assay -Less distance travelled and fewer rearing events in OFT**		**Increased anxiety in OFT**			**Reduced PPI**
		VTA-Cre	11- 13 weeks	ND socialbility in 3 chamber social approach		**Reduced rearing in OFT**		ND OFT			**Increased percentage of PPI of startle response to 120 dB sound with 74 or 78 dB prepulse**
Nunes and Kuner, 2018	*Scn2a :OB GC - shRNA*		49 to 63 days						**Reduced speed in odor discrimination**		

Paper	*Model*	Iteration of Conditional Model	Mice Age	Social	Learning/Memory	Motor	Repetitive Behaviors	Anxiety/Hyperactivity	Olfaction	Seizures	Other
Li et al., 2023	*Scn2a ASO*		2-3 months	**-Impaied social approcah ssay** **- Increased social avoidiance in 3-chamber assay**		**Increased locomotion in OFT**	**Impaired nest building behavior**	**-Decreased aniexty and ability to assess risk in in EPM** **- Increased anxiety-like behaviros in OFT**		-No spontaneous seziures or detecable ictal features **- Augmented seziure response to PTZ treatment**	
